# Activation of Toll-Like Receptor 7 Signaling Pathway in Primary Sjögren's Syndrome-Associated Thrombocytopenia

**DOI:** 10.3389/fimmu.2021.637659

**Published:** 2021-03-09

**Authors:** Shuo Zhang, Jingge Qu, Li Wang, Mengtao Li, Dong Xu, Yan Zhao, Fengchun Zhang, Xiaofeng Zeng

**Affiliations:** ^1^Department of Rheumatology and Clinical Immunology, Peking Union Medical College Hospital, Chinese Academy of Medical Sciences & Peking Union Medical College, Beijing, China; ^2^State Key Laboratory of Complex Severe and Rare Diseases, Peking Union Medical College Hospital, Chinese Academy of Medical Sciences & Peking Union Medical College, Beijing, China; ^3^National Clinical Research Center for Dermatologic and Immunologic Diseases (NCRC-DID), Ministry of Science & Technology, Beijing, China; ^4^Key Laboratory of Rheumatology and Clinical Immunology, Ministry of Education, Beijing, China

**Keywords:** primary Sjögren's syndrome, thrombocytopenia, B lymphcytes, Toll-like receptor 7, high-throughput nucleotide sequencing

## Abstract

**Objectives:** To identify the importance of the Toll-like receptor (TLR) pathway using B cell high-throughput sequencing and to explore the participation of the TLR7 signaling pathway in primary Sjogren's syndrome (pSS)-associated thrombocytopenia in patient and mouse models.

**Methods:** High-throughput gene sequencing and bioinformatic analyses were performed for 9 patients: 3 patients with pSS and normal platelet counts, 3 patients with pSS-associated thrombocytopenia, and 3 healthy controls. Twenty-four patients with pSS were recruited for validation. Twenty-four non-obese diabetic (NOD) mice were divided into the TLR7 pathway inhibition (CA-4948), activation (Resiquimod), and control groups. Serum, peripheral blood, bone marrow, and submandibular glands were collected for thrombocytopenia and TLR7 pathway analysis.

**Results:** Seven hub genes enriched in the TLR pathway were identified. Compared to that in control patients, the expression of interleukin (IL)-8 and TLR7 pathway molecules in B-cells was higher in patients with pSS-associated thrombocytopenia. Platelet counts exhibited a negative correlation with serum IL-1β and IL-8 levels. In NOD mice, CA-4948/Resiquimod treatment induced the downregulation/upregulation of the TLR7 pathway, leading to consistent elevation/reduction of platelet counts. Megakaryocyte counts in the bone marrow showed an increasing trend in the Resiquimod group, with more naked nuclei. The levels of IL-1β and IL-8 in the serum and submandibular gland tissue increased in the Resiquimod group compared with that in CA-4948 and control groups.

**Conclusion:** pSS-associated thrombocytopenia may be a subset of the systemic inflammatory state as the TLR7 signaling pathway was upregulated in B cells of patients with pSS-associated thrombocytopenia, and activation of the TLR7 pathway led to a thrombocytopenia phenotype in NOD mice.

## Introduction

Primary Sjögren's syndrome (pSS) is a systemic rheumatic disorder characterized by lymphocytic infiltration of the exocrine glands with or without multiple extra-glandular involvement (1). Thrombocytopenia, a hematological manifestation of pSS, is present in 7.8% of patients at the time of pSS diagnosis (2). pSS-associated thrombocytopenia seriously affects patients' quality of life and life expectancy and poses challenges in the clinical management.

The pathogenetic mechanisms of pSS-associated thrombocytopenia have not been fully elucidated. Previous study revealed the potential role of elevated plasma P-selectin autoantibodies in the pathogenesis of thombocytopenia in pSS patients (3). Current studies have revealed that B cells play an important role in the pathogenesis of pSS (4). While in patients with pSS associated severe thrombocytopenia, the expression of FcγRIIb on B cells was significantly decreased. After high-dose methylprednisolone pulse therapy, the platelet count was significantly increased, with the taised expression of FcγRIIb on memory B cells (5). It suggested that the humoral immune response in pSS associated thrombocytopenia. Moreover, studies have also revealed that innate immune response disorders are associated with both pSS and immune thrombocytopenia, including the Toll-like receptor (TLR) 7 signaling pathway (6, 7). TLRs play a critical role in initiating innate inflammatory responses; they can recognize a range of RNA and DNA molecules from viruses and self-antigens leading to the production of cytokines and immune cell responses (8). In patients with pSS, previous studies revealed that TLR 7 driven loss of tolerance in pSS (9). While the stimulation of TLR-7 led to more naïve B cells, less preswitched memory B cells, and fewer IL-10 positive preswitched memory B cells, with increased amounts of several cytokines (10). However, their underlying mechanism remains unclear.

Therefore, it is crucial to understand the precise molecular mechanisms of B cells in pSS-associated thrombocytopenia and thus develop effective therapeutic strategies. In the present study, the differentially expressed genes (DEGs) in B cells in pSS-associated thrombocytopenia were screened based on high-throughput sequencing of the whole exosome. We then explored whether and how the TLR 7 signaling pathway participates in the pathogenesis of pSS-associated thrombocytopenia in patients and in a mouse model of pSS. This study provides novel insights into pSS-associated thrombocytopenia and identifies potential biomarkers and targets for future therapeutic strategies.

## Materials and Methods

### Participants

Patients with pSS were enrolled in this study, including patients with pSS-associated thrombocytopenia and patients with pSS without thrombocytopenia. The diagnosis of pSS was defined according to the 2016 American College of Rheumatology (ACR)-European League against Rheumatism (EULAR) classification criteria (11). Thrombocytopenia was defined as platelet count < 100 × 10^9^/L. All the patients were newly diagnosed without any treatment. Patients with other connective tissue diseases (CTD), other hematological disorders, any kind of acute or chronic infection, cancer, familial, viral, or drug-induced thrombocytopenia were excluded. Nine subjects were used for RNA-sequencing: three patients with pSS and normal platelet counts, three patients with pSS characterized by thrombocytopenia, and three gender- and age-matched healthy controls without the history of any rheumatological conditions. Twenty-four patients with pSS were used for validation, 12 patients with pSS-associated thrombocytopenia, and 12 patients with pSS without thrombocytopenia. All patients were enrolled from the Peking Union Medical College Hospital (PUMCH). The present study was approved by the Ethics Committee of PUMCH (No. JS-1870). All patients provided written informed consent for the collection of blood samples and processing of their personal data for clinical research purposes.

### Cell Isolation and RNA Isolation

Peripheral blood mononuclear cells were isolated from heparinized peripheral blood by Ficoll-Paque density gradient centrifugation. B-lymphocytes were freshly isolated by magnetic cell sorting according to the manufacturer's instructions provided with the B-lymphocyte sorting kit (BD Biosciences, San Diego, USA). Cells were lysed in TRIzol, and total RNA was isolated. RNA concentrations were assessed with an Agilent 2100 Bioanalyzer (Agilent RNA 6000 Nano Kit), and RNA integrity was measured by capillary electrophoresis; all samples had a RIN-score > 7.0.

### High Throughput Sequencing

RNA-sequencing (RNA-seq) was performed by the Beijing Genomics Institute (BGI). The total mRNA was enriched by oligodT selection or rRNA depletion. After the mRNA was extracted, concentrated, and sheared into fragments, complementary DNA (cDNA) was synthesized using N6 random primers, followed by purification of fragments, terminal repair, polyA-tailing, and ligation of adapters. The raw data from a chain-specific library were obtained by amplification using real-time polymerase chain reaction (RT-PCR). RNA-seq was performed based on the BGISEQ- 500 platform. The internal software SOAPnuke was used to filter reads. After filtering, the remaining reads were called “clean reads” and stored in FASTQ format (12). Furthermore, the clean reads were mapped to references using Bowtie 2 (13). Finally, the gene expression level was calculated using RSEM (14). For the final report, genes were annotated according to the National Center for Biotechnology Information and Ensembl genome databases after data were cleaned according to alignment, assembly, and qualification.

### Identification of DEGs

Gene expression profiles were analyzed for patients with pSS without thrombocytopenia, patients with pSS-associated thrombocytopenia, and healthy controls. DEGs in group 1 (pSS-associated thrombocytopenia patients vs. healthy controls) and group 2 (pSS-associated thrombocytopenia patients vs. pSS patients without thrombocytopenia) were identified using the limma R package (15). Then, based on the “edge R” packages in R with an absolute fold change (log 2) > 2, the false discovery rate (FDR) was adjusted to a *P*-value < 0.01 to correct for the statistical significance of multiple experiments. Heat maps and volcano maps were generated individually using the gplots and pheatmap package (1.0.8) within R, while VennPlex (16) was used to create Venn diagrams and to determine overlapping genes between group 1 and 2.

### Kyoto Encyclopedia of Genes and Genomes and Gene Ontolog Enrichment Analyses of DEGs

To determine the function represented in DEGs, we used the Database for Annotation, Visualization and Integrated Discovery (DAVID) to perform a functional and enrichment analysis of the DEGs using the GO and KEGG analysis R package (17). Subsequently, to provide a visible graphic that represents the interactions of DEGs and relative KEGG pathways, the gene pathway network was established and visualized using the Biological Networks Gene Oncology tool (BiNGO) (version 3.0.3) plugin of Cytoscape (18). Meanwhile, the KEGG mapper was used to understand high-level functions and biological systems from large-scale molecular datasets generated by high-throughput experimental technologies. In the GO analysis and KEGG pathway analysis, a *P*-value of <0.05 was considered statistically significant.

### Protein-Protein Interaction Network Analysis

We used the PPI network provided by the Retrieval of Interacting Genes (version 10.0) (19) online database. Analyzing the functional interactions between proteins may provide insights into the mechanisms of generation or development of diseases. In this study, the PPI network of DEGs was constructed using the STRING database. An interaction with a combined score > 0.4 was considered statistically significant. The network was displayed using Cytoscape (version 3.4.0) (20). We then used the Cytoscape app plug-in Molecular Complex Detection (MCODE) (version 1.4.2) for clustering a given network based on topology to identify densely connected regions (21). The criteria for selection were as follows: MCODE score > 6, degree cut-off = 2, node score cut-off = 0.2, Max depth = 100, and k-score = 2.

### Hub Gene Selection and Analysis

The Cytoscape app Cytohubba (version 0.1) was used to identify the hub genes (22), and the genes with the highest degree were selected. The association and biological functions among nodes with the highest interaction degree were analyzed with GenCLiP 2.0 (23), which enables functional annotation and molecular network construction of genes based on published literature.

### Animal Study

An animal study was performed in accordance with the principles established by the revised Dutch Act on Animal Experimentation (1997) and was approved by the Ethics Committee of the Peking Union Medical Collage Hospital. Non-obese diabetic (NOD) mice were obtained from the Institute of Laboratory Animal Science, Chinese Academy of Medical Science. Twenty-four NOD mice were randomly allocated to three treatment groups. CA-4948 (50 μg/mouse, once a day) (HY-135317, MedChem Express, Princeton, USA), Resiquimod (50 nmol/mouse, once a week) (HY-13740, MedChem Express, Princeton, USA), or saline was administered by gavage at 8 weeks of age. Peripheral blood was obtained and analyzed every 5 days. After 5 weeks, the submandibular glands and bone marrow were harvested. Platelet counts in the peripheral blood, megacaryocyte (MK) counts and morphology in the bone marrow, TRL pathway, and platelet-related markers in the serum, peripheral blood cells, and submandibular glands were analyzed.

### ELISA

Plasma levels of cytokines and platelet-related biomarkers were quantified using enzyme-linked immunosorbent assays (ELISA) in validation cohort as well as in animal study, according to the manufacturer's instructions. The following ELISA kits were used: IL-1β (mouse cat. SEA563Mu, human cat. SEA563Hu, Boster, Wuhan, China), IL-8 (mouse cat. ml058632, Shanghai Enzyme-linked Biotechnology Co., Ltd., Shanghai, China; human cat. SEA080Hu, Boster, Wuhan, China), thrombopoietin (TPO) (mouse cat. SEA135Mu, human cat. SEA135Hu, Boster, Wuhan, China), and Megakaryocyte Colony Stimulating Factor (MK-CSF) (mouse cat. SEA090Mu, human cat. SEA090Hu, Boster, Wuhan, China).

### RT-PCR

Single-stranded cDNAs were synthesized using a SYBR PrimeScript RT-PCR kit (TaKaRa Bio Inc., Japan), and PCR amplification was performed for real-time measurement of transcription in validation cohort as well as in animal study. The expression level was calculated by using 2^−ΔΔCT^ methods. Data were analyzed using the SDS 2.4 software (Applied Biosystems, Foster City, USA). The primer sequences are shown in Supplementary Table 1.

### Immunohistochemistry

Immunohistochemical staining for IL-1β, IL-8, TPO, and MK-CSF in the submandibular glands of NOD mice was performed. Tissue slides were probed with primary antibodies at indicated concentrations overnight at 4°C, followed by incubation with HRP-conjugated secondary antibody at room temperature for 1 h. Antibodies were applied at the following concentrations: IL-1β (cat. ab9722, Abcam, Cambridge, UK) 1:500 dilution, IL-8 (cat. A00423-1, Boster, Wuhan, China) 1:200 dilution, TPO (cat. ab203057, Abcam, Cambridge, UK) 1:500 dilution, and MK-CSF (cat. ab233387, Abcam, Cambridge, UK) 1:1,000 dilution. Chromogenic 3,3-diaminobenzidine (DAB) substrate was added to visualize the expression of the target proteins. Images were taken using a digital microscope color camera (Leica Microsystems, Tokyo, Japan).

### Western Blotting

Western blotting was performed to detect the protein levels of IL-1β, IL-8, TPO, and MK-CSF in the submandibular glands of the NOD mice. The submandibular glands were lysed, protein concentrations were measured, and identical amounts of protein were subjected to 10% sodium dodecyl sulfate-polyacrylamide gel electrophoresis (SDS-PAGE). The proteins were transferred to a polyvinylidene fluoride (PVDF) filter. After blocking with blocking solution, the PVDF filter was incubated with rabbit anti-IL-1β antibody (cat. ab9722, Abcam, Cambridge, UK) (1:1,000 dilution), rabbit anti-IL-8 antibody (cat. ab18672, Abcam, Cambridge, UK) (1:1,000 dilution), mouse anti-TPO antibody (cat. ab203057, Abcam, Cambridge, UK) (1:1,000 dilution), rabbit anti-MK-CSF antibody (cat. ab216884, Abcam, Cambridge, UK) (1:1,000 dilution), rabbit anti-TLR7 antibody (cat. ab24184, Abcam, Cambridge, UK) (1:1,000 dilution), rabbit anti-MyD88 antibody (cat. ab2064, Abcam, Cambridge, UK) (1:1,000 dilution), mouse anti-IRAK4 antibody (cat. ab119942, Abcam, Cambridge, UK) (1:1,000 dilution), rabbit anti-TRAF6 antibody (cat. ab33915, Abcam, Cambridge, UK) (1:1,000 dilution), mouse anti-NF-κB p65 antibody (cat. 6956, CST, Danvers, USA) (1:1,000 dilution), or rabbit anti-β-actin antibody (cat. ab228387, Abcam, Cambridge, UK) (1:1,000 dilution) at 4°C overnight. The filter was then washed and incubated with mouse or rabbit anti-IgG coupled with horseradish peroxidase (HRP) as the secondary antibody. The signal was detected using a ChemiDoc MP chemiluminescence system (Bio-Rad, USA).

### Statistical Analysis

Statistical analyses were performed using SPSS 25.0 software (SPSS Inc., Chicago, IL, USA) and R statistical software version 3.4.3 (http://www.R-project.org/). Data are presented as the mean ± standard deviation (SD) for normally distributed continuous variables or the number and proportion (%) for categorical variables. Data were compared between groups using Student's *t*-test for continuous variables. A *p*-value < 0.05 was considered to be statistically significant.

## Results

### Subject Characteristics

The study population characteristics are shown in Table 1. The mean age ± SD of the RNA-sequencing cohort and validation cohort pSS patients with thrombocytopenia meeting the inclusion criteria and the ones without thrombocytopenia (control group) were 54.5 ± 13.1 years and 56.5 ± 13.9, respectively. All subjects were women. The average platelet counts were 57.7 ± 24.5 in the pSS patients with thrombocytopenia group, which was significantly lower than that in the control group (230.0 ± 36.8) (*p* < 0.001). For all the samples, the peripheral blood leucocyte and erythrocytes counts were within the normal range.

**Table 1:** Study population characteristics.

	**Subjects for RNA-sequencing (*****n*** **= 9)**			**Subjects for validation (*****n*** **= 24)**	
	**Pss (*n* = 3)**	**pSS associated thrombocytopenia (*n* = 3)**	**Healthy controls (*n* = 3)**	**pSS (*n* = 12)**	**pSS associated thrombocytopenia (*n* = 12)**
**Demographics**					
Age	54.0 ± 8.2	55.0 ± 14.1	53.3 ± 6.0	54.8 ± 11.8	54.4 ± 12.7
Female sex	3 (100%)	3 (100%)	3 (100%)	12 (100%)	12 (100%)
**Symptoms**					
Xerostomia	3 (100%)	3 (100%)	–	4 (33%)	9 (75%)
Xerophthalmia	3 (100%)	3 (100%)	–	4 (33%)	7 (58%)
Arthralgia	2 (66.6%)	1 (33.3)	–	8 (66.6%)	3 (25%)
**Auto-antibodies**					
ANA > 1:100	2 (66.6%)	3 (100%)	–	8 (66.6%)	9 (75%)
Anti-Ro/SSA antibodies	2 (66.6%)	2 (66.6%)	–	10 (83.3%)	9 (75%)
Anti- La/SSB antibodies	0	1 (33.3%)	–	8 (66.6%)	3 (25%)
**Other lab values**					
ESR	53.3 ± 26.2	40.3 ± 15.4	–	104.3 ± 78.4	40.9 ± 42.1
IgG	22.0 ± 3.3	19.0 ± 1.6	–	19.5 ± 7.2	12.8 ± 6.1

*pSS, primary Sjögren's syndrome; ESR, erythrocyte sedimentation rate; IgG, immunoglobulin G*.

#### Nineteen DEGs Were Identified in pSS-Associated Thrombocytopenia

**Figure 1:**
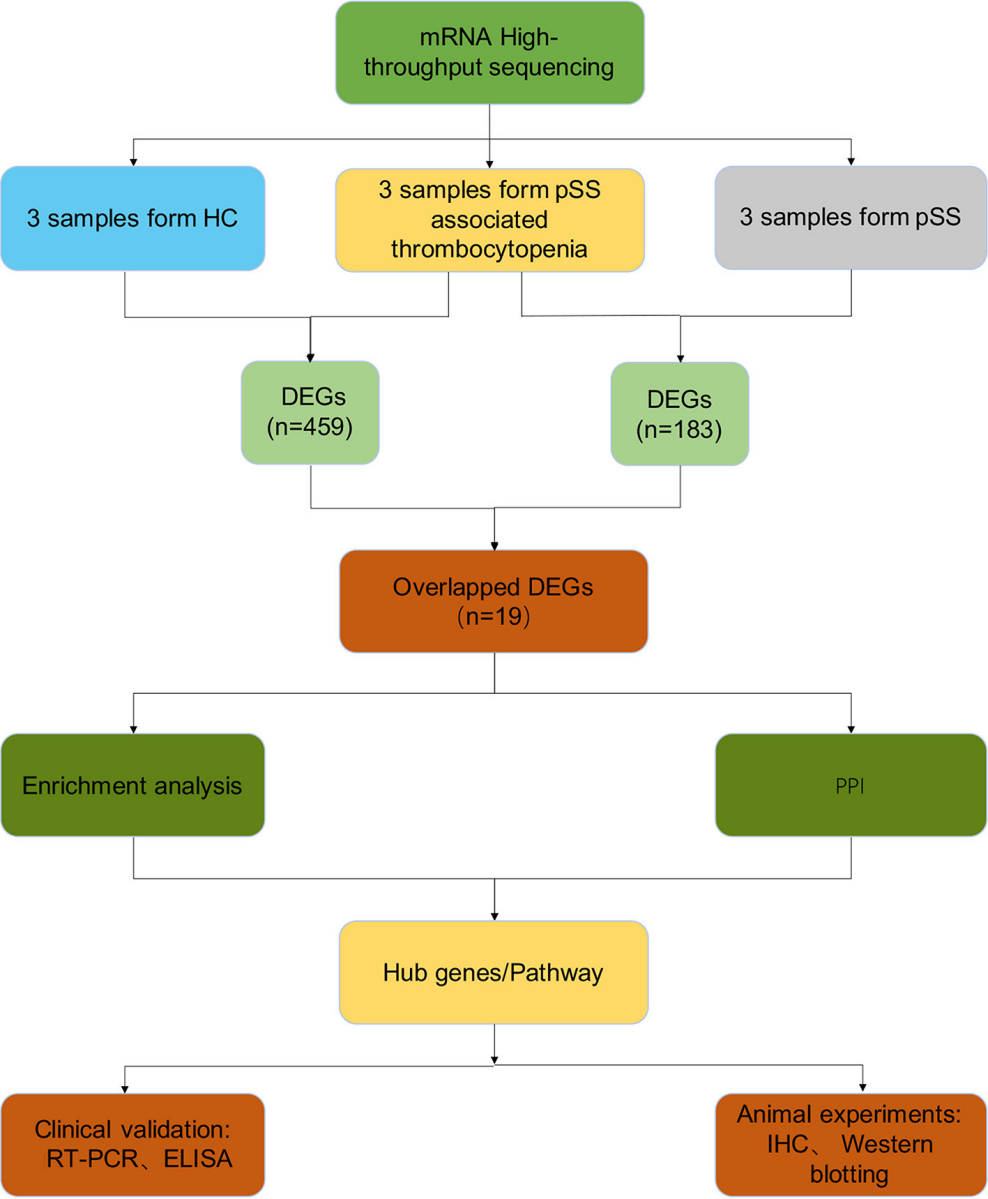
Overview of the study design. HC, healthy control; pSS, primary Sjögren's syndrome; DEGs, differentially expressed genes; PPI, protein-protein interaction; RT-PCR, real-time polymerase chain reaction; ELISA, enzyme-linked immunosorbent assays; IHC, immunohistochemical.

A general overview of the study design is shown in Figure 1. After standardization of the high-throughput sequencing results, 459 DEGs between health controls and pSS associated thrombocytopenia were identified (2 upregulated and 457 downregulated) (Supplementary Figures 1A,C). These 459 DEGs contributing to the development of pSS, as well as thrombocytopenia in pSS. To further confirm the DEGs specified in pSS-associated thrombocytopenia, 183 DEGs (31 upregulated and 151 downregulated) from group 2 were identified to be associated with thrombocytopenia in pSS (Supplementary Figures 1B,D). Therefore, the overlap between the two groups contained 19 genes representing the DEGs specified in pSS-associated thrombocytopenia, as shown in the Venn diagram (Supplementary Figure 1E). All the DEGs were upregulated in both group 1 and group 2 (Supplementary Table 2).

#### Five GO Terms Were Identified by Enrichment Analyses of the 19 DEGs

**Figure 2:**
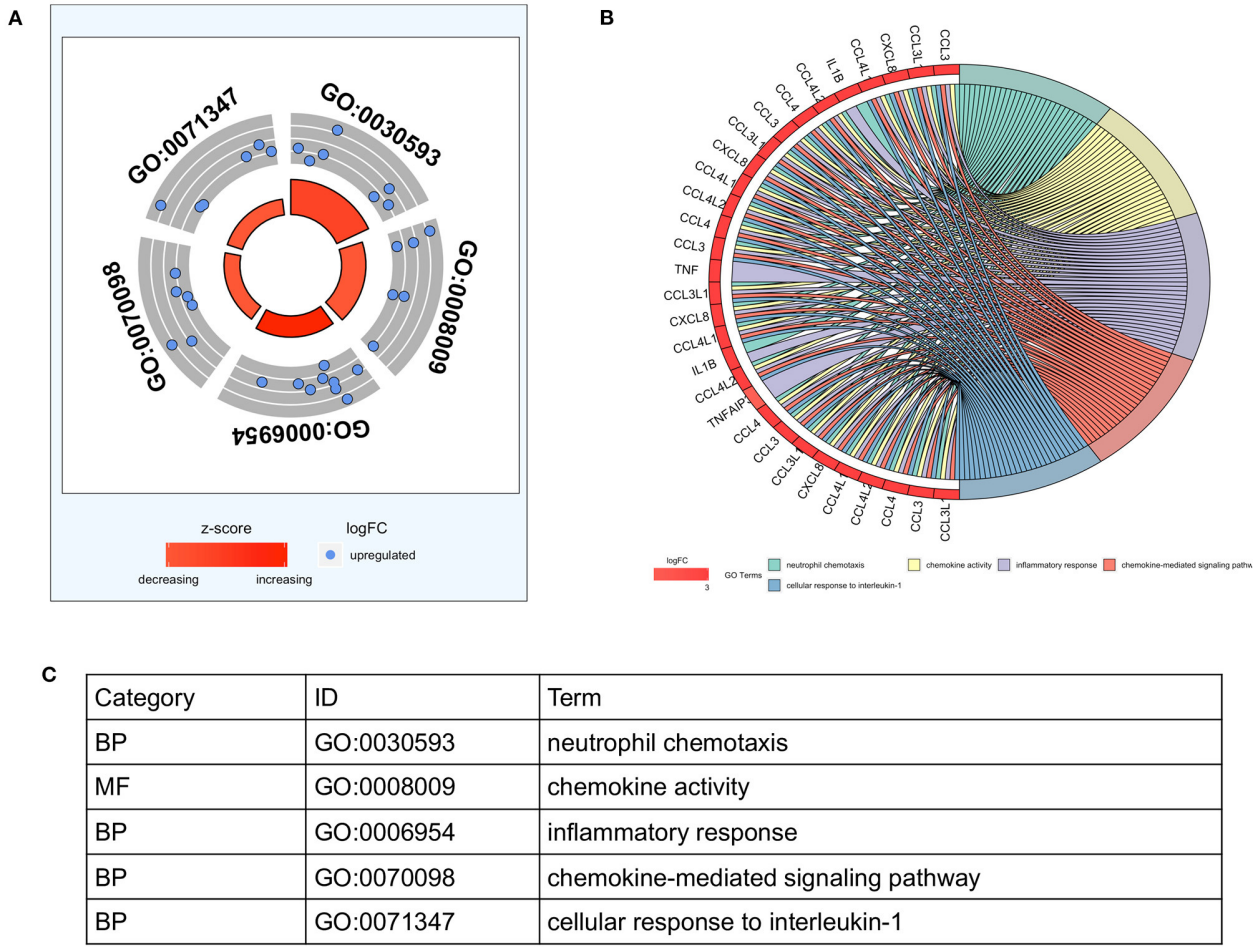
Functional enrichment analysis of 19 differentially expressed genes (DEGs) in pSS associated thrombocytopenia. **(A)** The outer circle represents the expression (log FC) of DEGs in each enriched GO (gene ontology) term: blue dots on each GO term indicate upregulated DEGs. The inner circle indicates the significance of GO terms (log10-adjusted *P*-values). **(B)** The circle indicates the correlation between the top 9 DEGs and their gene ontology terms. **(C)** The distribution of DEGs in significant GO terms. DEGs, differentially expressed genes; pSS, primary Sjögren's syndrome; FC, fold change; BP, biological process; MF, molecular function.

To analyze the biological classification of DEGs, functional and pathway enrichment analyses were performed using DAVID. Gene ontology analysis showed 5 GO terms (neutrophil chemotaxis, chemokine activity, inflammatory response, chemokine-mediated signaling pathway, cellular response to interleukin-1) with significant differences (*p* < 0.05), and the highest GO biological process was “GO:0030593 neutrophil chemotaxis” (Figure 2).

The GO Chord plot showed the top 9 DEGs with their related GO terms, which were CCL3, CCL4, CCL3L1, CCL4L1, CCL4L2, CXCL8 (IL-8), IL1B, TNF, and TNFAIP3 (Figure 2B). KEGG pathway analysis revealed that the upregulated DEGs were mainly enriched in the Toll-like receptor (TLR) signaling pathway, Salmonella infection, viral protein interaction with cytokine and cytokine receptor, NF-kappa B signaling pathway, human cytomegalovirus infection, cytokine-cytokine receptor interaction, rheumatoid arthritis, IL-17 signaling pathway, chemokine signaling pathway, chagas disease, cytosolic DNA-sensing pathway, and AGE-RAGE signaling pathway in diabetic complications (Supplementary Figure 2A). The KEGG pathway network of the upregulated DEGs is shown in Supplementary Figure 2B. CXCL8, IL1B, and TNF were the top 3 DEGs involved in all 11, 10, and 9 pathways, respectively. Meanwhile, the TLR signaling pathway was the most significant pathway with the highest number of DEGs in the KEGG enrichment analysis (Supplementary Figure 2B). Through the KEGG mapper, we found that all the DEGs enriched in the Toll-like receptor pathway were for inflammatory cytokines (Supplementary Figure 2C).

#### PPI Network Analysis, Module Analysis, and Hub Gene Identification

The PPI network of DEGs was constructed (Supplementary Figure 3A). A total of 13 DEGs from 19 candidate DEGs were filtered into a network consisting of 37 interaction pairs among these 13 nodes. In addition, the most significant module was obtained based on the MCODE analysis in Cytoscape (Supplementary Figure 3B). This module consisted of 7 nodes (TNF, CXCL8, CCL4L1, IL1B, CCL3, CCL3L1, and CCL4) and 21 edges. As the functional enrichment analysis showed above (Supplementary Figure 2), all 7 DEGs were enriched in the TLR signaling pathway.

#### Identification and Analysis of the Hub Genes

The Cytoscape cytoHubba Network Analyzer plug-in selected 10 hub genes from the PPI network by identifying the top 10 nodes ranked by degree. Out of the 10 nodes investigated, 7 were significant and had a degree ≥ 700. Coincidentally, they were exactly the same 7 genes obtained from the MCODE analysis. TNF, CXCL8, CCL4L1, IL1B, CCL3, CCL3L1, and CCL4 were further confirmed by the CytoHubba network of hub genes. TNF had 7 co-interacting genes in the network. TNF and IL1B showed 18,190 co-citations in the network out of a total of 22,150 in the literature. TNF and CXCL8 showed 6,312 of 22,150 co-citations. IL1B and CXCL8 showed 4,474 of 20,350 co-citations (Supplementary Figure 3).

### Increased Expression of Serum and B-Cell IL-8 in pSS Patients With Thrombocytopenia

Relative expression levels of these 7 hub genes in the B cells of patients with pSS were further determined by RT-PCR in the validation cohort. Compared to that in pSS patients without thrombocytopenia, the relative expression of IL-8 mRNA and CCL3L in B-lymphocytes in patients with pSS-associated thrombocytopenia was increased. Meanwhile, the pSS-associated thrombocytopenia group displayed a trend toward increasing levels of TNFα expression. However, no differences were observed in the relative levels of IL-1β or TNFα mRNAs between these two groups. Some potential serum biomarkers of thrombocytopenia were further evaluated by ELISA. The PSS-associated thrombocytopenia group had significantly elevated levels of IL-8 (196.5 ± 73.4 vs. 137.2 ± 52.9, *p* = 0.033) and IL-1β (158.5 ± 23.6 vs.130.8 ± 38.7, *p* = 0.047) compared to the pSS without thrombocytopenia group.

### Upregulation of TLR7 Pathway in pSS-Associated Thrombocytopenia Compared to pSS Without Thrombocytopenia

**Figure 3:**
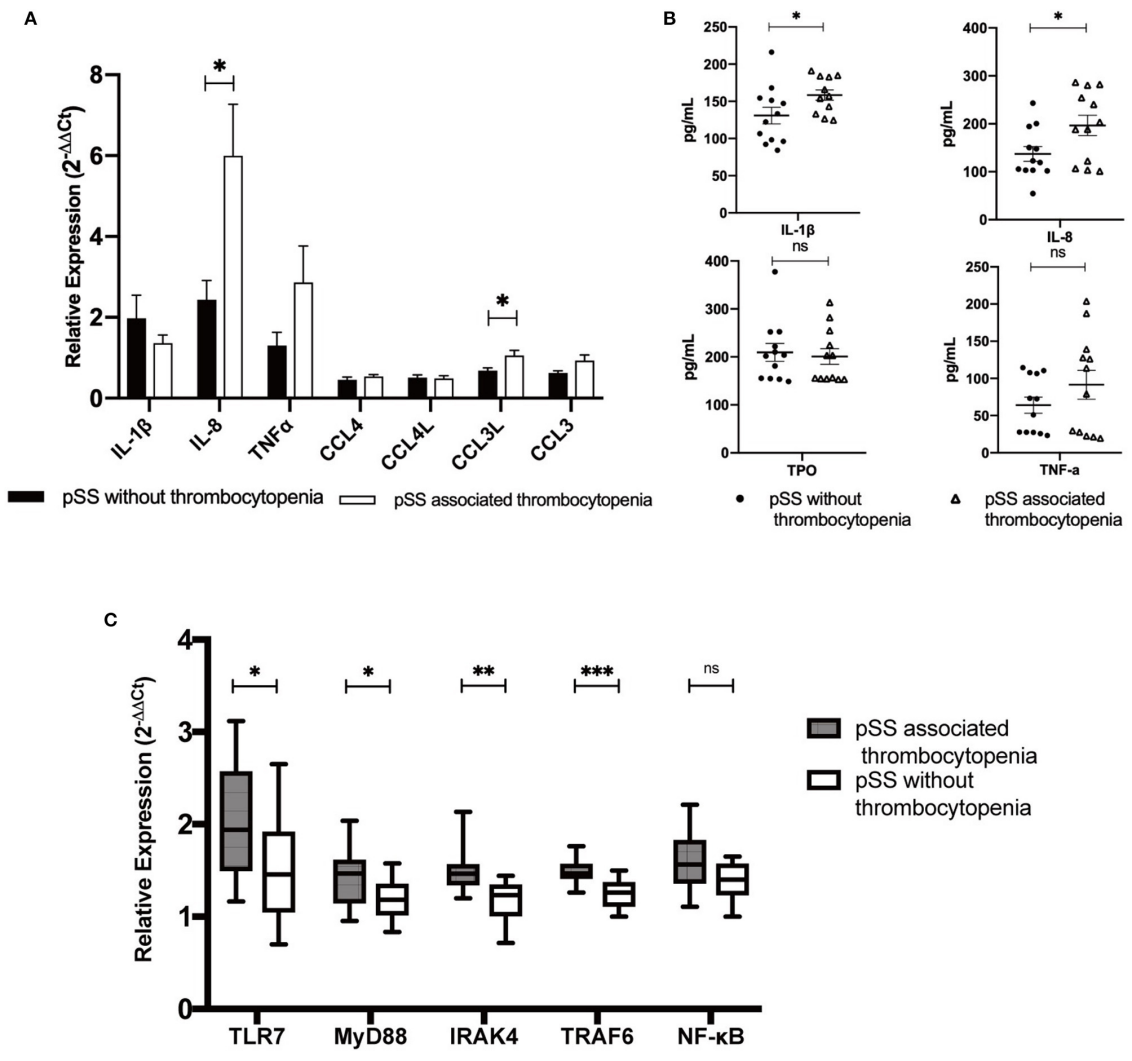
Hub genes expression, upregulation of Toll-like receptor (TLR) 7 and its downstream signaling molecules in B-cell and serum in pSS patients with or without thrombocytopenia in the validation cohort. **(A)** mRNA relative expression levels of 7 hub genes in B cells determined by real-time polymerase chain reaction (RT-PCR). Compared to that in pSS patients without thrombocytopenia, the relative expression of interleukin (IL)-8 mRNA and CCL3L in B-lymphocytes in patients with pSS-associated thrombocytopenia was increased. **(B)** Serum level of IL-1β, IL-8, thrombopoietin (TPO) and TNFα. **(C)** Upregulation of TLR7 and its downstream signaling molecules in patients with pSS associated thrombocytopenia compared to those in pSS patients without thrombocytopenia (*p < 0.05, **p < 0.01, ***p < 0.001; ns, not significant).

A previous bioinformatics analysis and validation study in patients with pSS revealed the TLR7 pathway as the most canonical pathways in pSS-associated thrombocytopenia compared to pSS patients without thrombocytopenia and healthy controls in the validation cohort. Prior studies have found a positive association between the TLR7 signaling pathway and pSS as well as ITP (6, 7). Thus, we detected TLR7 and its downstream signaling molecules (24, 25) in B cells of patients with pSS-associated thrombocytopenia compared to pSS patients without thrombocytopenia in the validation cohort. Compared with that in the control group, the expression of TLR7, MyD88, IRAK4, and TRAF6 increased significantly in the B cells of patients with pSS associated thrombocytopenia (*p* < 0.05) (Figure 3).

### Serum and B-Cell IL-8 Expression Level Correlated to Platelet Count in pSS Patients With Thrombocytopenia

The correlation between platelet count level and serum and B-cell cytokine expression levels was analyzed in the validation cohort. Notably, platelet counts negatively correlated with serum IL-1β (*r* = −0.409, *p* = 0.047) and IL-8 (*r* = −0.415, *p* = 0.044). In B cells, the relative expression of IL-8 (*r* = −0.479, *p* = 0.018) and CCL3L (*r* = −0.588, *p* = 0.003) mRNA also negatively correlated with platelet count. No correlation was found between platelet count and serum TNFα level or any other hub gene mRNA expression in the B cells (*p* > 0.05).

### Stimulating the TRL7 Pathway Induced Platelet Decrease in NOD Mice

**Figure 4:**
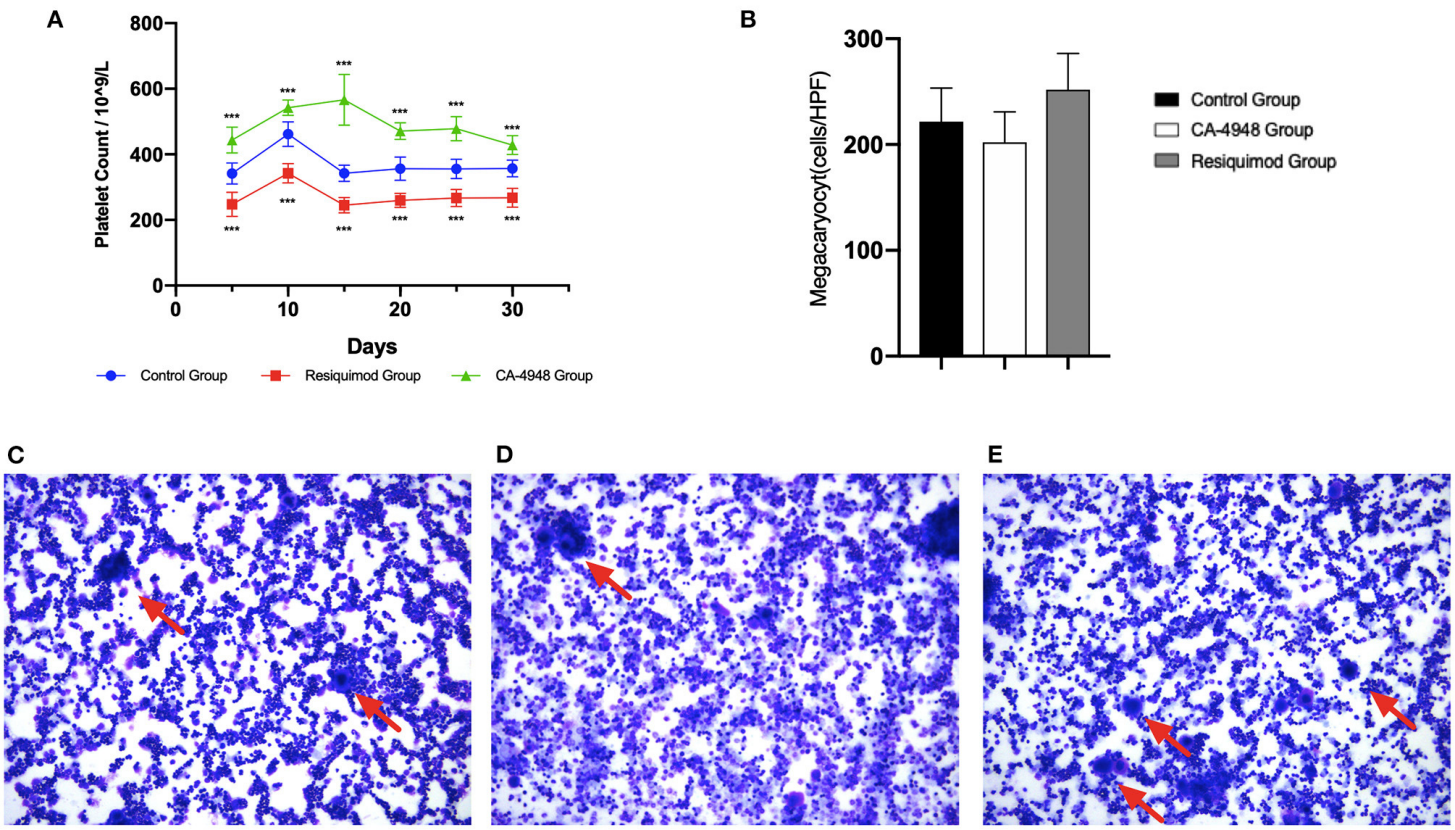
Effects of Tol-like Receptor (TLR) pathway activation or inhibition on platelet and megakaryocyte in bone marrow in Non-obese diabetic (NOD) mice. **(A)** Platelet counts changes after CA-4948 (*n* = 8)/Resiquimod (*n* = 8)/saline (*n* = 8) treatment in NOD mice. After 5 days of treatment, consistent significant increase of platelet counts were observed in the CA-4948 group compared with the ones in the control group. **(B)** Megakaryocyte (MK) counts after CA-4948/Resiquimod/saline treatment in NOD mice. **(C)** MK morphology in control group. **(D)** MK morphology in CA-4948 group. **(E)** MK morphology in Resiquimod group. More naked nucleus MK were observed in the Resiquimod group, although without statistical significance. Red Arrow, MK (***p < 0.001).

We further investigated whether intervention of the TLR7 pathway may affect platelet count and induce pSS-associated thrombocytopenia-like manifestations in pSS model mice. The groups treated with the TLR7 pathway inhibitor (CA-4948) or agonist (Resiquimod) were compared with the saline-treated control group. Platelet counts were determined every 5 days after treatment from 8 to 13 weeks. After 5 days of treatment, consistent significant differences in platelet counts among different groups were observed (Figure 4A). Compared to the ones in the saline control group, the platelet counts in the CA-4948 group were significantly increased (*p* < 0.001), while the counts in the Resiquimod group were decreased (*p* < 0.001).

### TRL7 Pathway Blockage and Stimulation Induced Megakaryocyte Changes in Bone Marrow of NOD Mice

The counts and morphology of megakaryocytes (MK) in the bone marrow of NOD mice were evaluated. Compared to that in the control group (221.6 ± 31.7), the amount of MK in the CA-4948 group decreased slightly (202.1 ± 28.7) (*p* = 0.217), while the amount of MK in the Resiquimod group increased (251.6 ± 34.3) (*p* = 0.088) but without statistical significance (Figure 4B). More naked nucleus MK were observed in the Resiquimod group than the other two groups (Figures 4C–E). MK counts negatively correlated with average platelet counts (*r* = −0.455, *p* = 0.025).

### TRL7 Pathway Intervention Induced Related Serum Marker Changes in NOD Mice

Serum biomarkers and downstream molecules of the TRL7 pathway were measured by ELISA and RT-PCR. To better understand the platelet count changes, TPO and MK-CSF were determined. Compared with the control group, treatment of NOD mice with CA-4948 led to a significant decrease in serum and peripheral blood mRNA expression of the TLR7 pathway molecules, IL-1β, IL-8, and MK-CSF levels and increase in serum TPO levels. However, the Resiquimod group showed the opposite trend. The serum levels of IL-1β, IL-8, and MK-CSF negatively correlated with average platelet counts (*r* = −0.475, −0.414, −0.442, respectively) (Supplementary Figure 4).

### TLR7 Pathway Stimulation Induced IL-8 Expression in the Submandibular Glands in NOD Mice, Which Increases During the Development of SS-Like Disease

**Figure 5:**
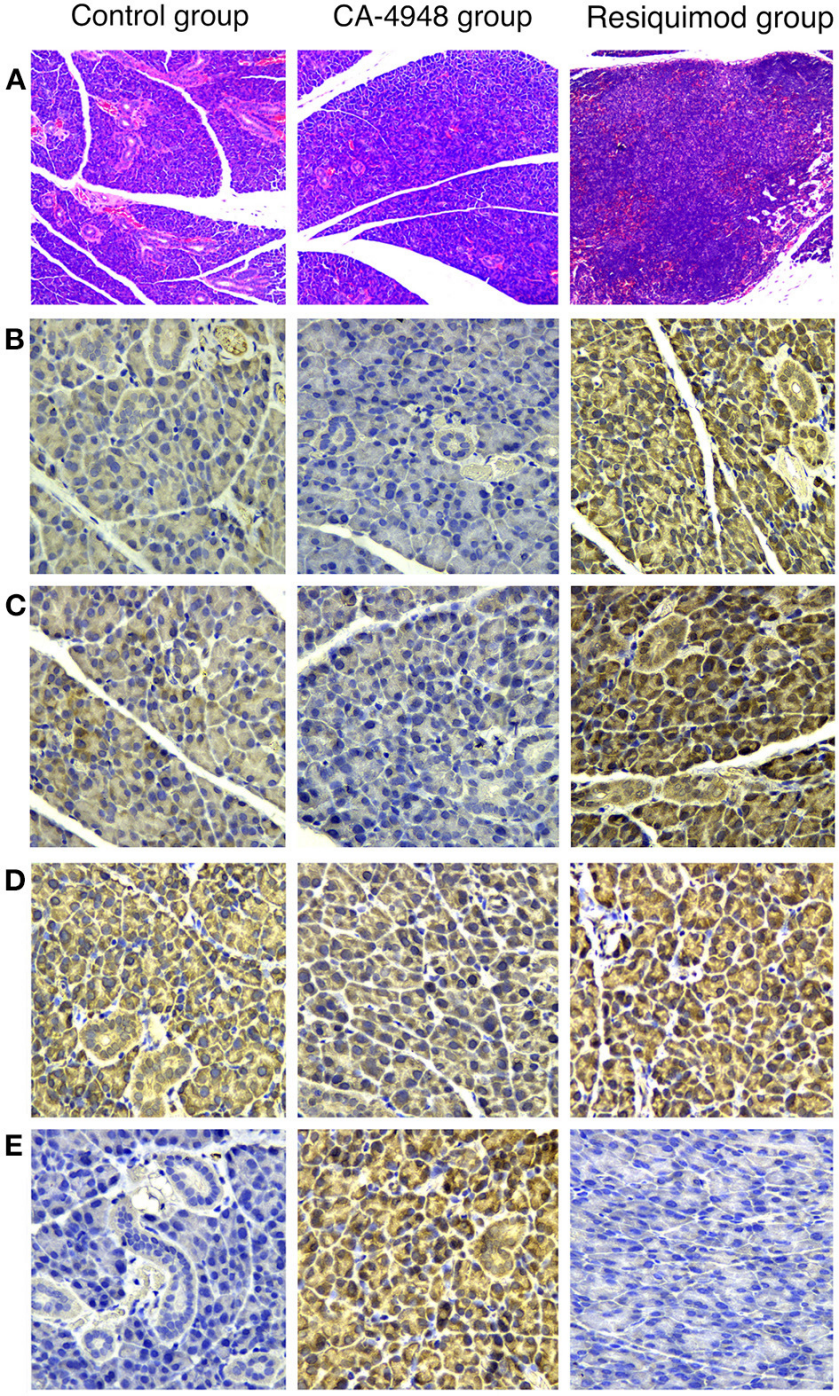
Hematoxylin and eosin-stained sections and Immunohistochemical (IHC) stained sections of the submandibular gland tissue of NOD mice. Submandibular gland was obtained at the age of 13 weeks, 5 weeks after intervention of CA-4948/Resiquimod/saline. **(A)** Infiltration of scattered lymphocytes was present in interstitial tissue (original magnification × 100). **(B)** IHC of Interleukin (IL)-1β (original magnification × 400). **(C)** IHC of IL-8 (original magnification × 400). **(D)** IHC of Megakaryocyte Colony Stimulating Factor (MK-CSF) (original magnification × 400). **(E)** IHC of thrombopoietin (TPO) (original magnification × 400). Expression levels of IL-1β, IL-8, and MK-CSF were stronger in the Resiquimod-treated group than in the saline control. However, decreased expression of IL-1β, IL-8, and MK-CSF and increased expression of TPO in the CA-4948 group were observed compared to that in the control group.

**Figure 6:**
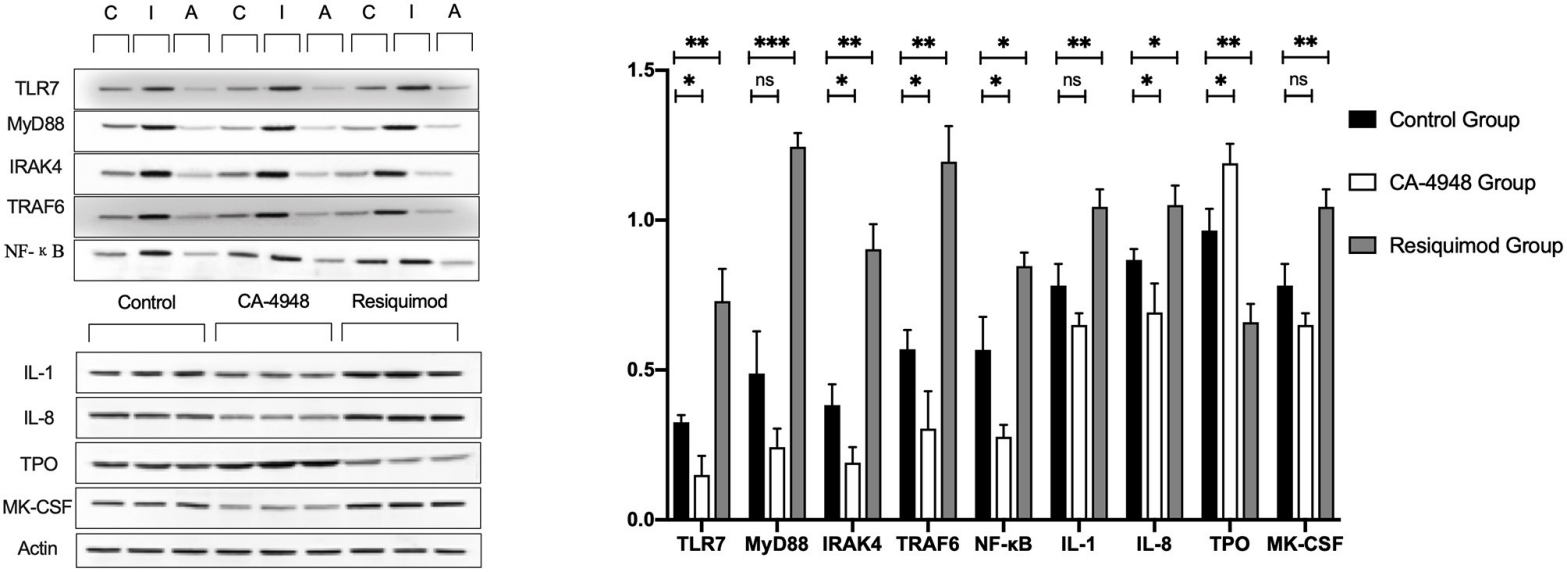
Western blot of TLR pathway and platelet-related markers of the submandibular gland tissue of NOD mice. Submandibular gland was obtained at the age of 13 weeks, after intervention of CA-4948/Resiquimod/saline for 5 weeks. C: control group treated with saline; I: inhibitor group (treated with CA-4948); A: activator group (treated with Resiquimod). Compared with saline control group, Resiquimod induced significantly increased expression of the TLR7 signaling pathway molecules, interleukin (IL)-1β, IL-8, and Megakaryocyte Colony Stimulating Factor (MK-CSF), and decreased expression of TPO in the submandibular glands. While CA-4948 group showed opposite expression trends of these molecules (*p < 0.05, **p < 0.01, ***p < 0.001; ns, not significant).

To confirm the effects of the TLR pathway intervention on submandibular glands, H&E - stained sections and IHC-stained sections were evaluated. Infiltration of lymphocytes in the interstitial tissue was observed in the salivary glands of controls at the age of 13 weeks, accompanied by ductal dilatation and scattered focal lymphocytic infiltration (Figure 5A). Expression levels of IL-1β, IL-8, and MK-CSF were stronger in the Resiquimod-treated group than in the saline control. However, decreased expression of IL-1β, IL-8, and MK-CSF and increased expression of TPO in the CA-4948 group were observed compared to that in the control group (Figures 5B–E). Quantitative western blot analyses revealed significantly increased expression of the TLR7 signaling pathway molecules, IL-1β, IL-8, and MK-CSF, and decreased expression of TPO in the submandibular glands of the Resiquimod-treated NOD mice (Figure 6).

## Discussion

Thrombocytopenia remains a serious systemic complication of pSS. However, the mechanisms involved remain unclear, which lead to challenges in the clinical management. The findings of this study indicate that TLR7 and its downstream signaling molecules are strongly expressed in patients with pSS-associated thrombocytopenia. This role of the TLR7 pathway has also been demonstrated in *in vivo* models of pSS.

Evidence confirms that lymphocytic disturbances play a significant role in SS, including ectopic germinal center formation and aberrations in cellular signaling (26). B cells are the dominant lymphocytes in severe salivary gland lesions (27). Given that B cell overactivity is a cardinal feature of pSS, as evidenced by the presence of profound hypergammaglobulinemia and several autoantibodies (28), direct or indirect targeting of B cells represents a therapeutic approach (29). In patients with pSS-associated thrombocytopenia, rituximab showed good efficacy and fair tolerance in a clinical observation study (30). Therefore, it is essential to screen for differences in gene expression in B cells of pSS-associated thrombocytopenia. Here, we exploited RNA-sequencing to provide in-depth transcriptional analysis of B cells from patients with pSS-associated thrombocytopenia, pSS without thrombocytopenia, and healthy controls. A total of 19 overlapped DEGs were identified between group 1 and group 2, and all DEGs were upregulated. In GO analysis, the DEGs were mainly enriched in the neutrophil chemotaxis, chemokine activity, inflammatory response, chemokine-mediated signaling pathway, and chemokine-mediated signaling pathway.

In our study, the TLR signaling pathway was the most significant pathway of pSS-associated thrombocytopenia, with the highest number of DEGs in the KEGG enrichment analysis. TLRs are an important part of the innate immune system and play an indispensable role in the pathogenesis of autoimmune diseases (8, 31). TLRs are an evolutionarily ancient family of pattern recognition receptors (PRRs) that recognize a wide range of pathogen-associated molecular patterns and activate a variety of immune cells, leading to the production of many immune-stimulatory cytokines and chemokines (8, 32). Several studies have demonstrated that aberrant activation of TLRs might result in unrestricted inflammatory responses, leading to the development of autoimmune diseases. The expression levels of TLR2-4 and TLR7 were higher in the labial salivary glands and/or in the cultured salivary gland epithelial cells in SS patients compared to controls (7, 33, 34). TLRs have also been shown to participate in thrombocytopenia in various diseases, including ITP and virus-induced thrombocytopenia (6, 35, 36).

Our present data demonstrated the increased expression of the TLR7 pathway molecules in B cells of patients with pSS-associated thrombocytopenia compared to pSS patients without thrombocytopenia. We also demonstrated that TLR7 activation could induce thrombocytopenia in a mouse model of pSS. TLR7 can be activated by endogenous host RNA and DNA. Then, it signals through the cytosolic adaptor MyD88, which associates with IRAK1/4, triggers TRAF6, and activates NF-κB to induce inflammatory cytokines and inflammatory responses (24). TLR7 was found to be significantly increased in some autoimmune diseases, including SLE and pSS (4, 6, 37). Yang et al. (6) also revealed that TLR7 contributes to autoantibody-mediated platelet destruction and correlates with disease activity in ITP. In this study, we showed that pSS-associated thrombocytopenia elicited a significantly stronger inflammatory response related to the TLR7 signaling pathway activation than in general patients with pSS, as indicated by increased inflammation, cytokine secretion, and NF-κB activity. Therefore, pSS-associated thrombocytopenia usually is usually correlated with a higher-grade inflammatory state and requires more aggressive treatment in clinical practice. Our findings suggest that pSS-associated thrombocytopenia might be a subset of pSS characterized by a systemic inflammatory state, which might be induced by stronger TLR7 pathway activation.

Another key finding from our analysis is the increased level of IL-8 and IL-1β expression in pSS-associated thrombocytopenia. IL-8 is a proinflammatory chemokine that belongs to the CXC family and induces neutrophil activation, binds to heparin, and is related to platelet factor 4 (38). Being a downstream signaling molecule of the TLR7 pathway, increase in IL-8 can reflect the activation of the TLR7 pathway and the pathogenesis leading to thrombocytopenia in pSS. Compared to healthy controls, there were no differences in serum IL-8 expression levels in patients with pSS (39). However, some studies revealed that IL-8 expression was significantly increased in induced tears and salivary glands of patients with pSS compared to healthy controls (40, 41). In healthy salivary gland epithelial cells, anti-Ro/SSA autoantibodies could stimulate the production of IL-8 (42). However, downregulating IL-8 levels would improve platelet recovery due to thrombocytopenia caused by myelodysplastic syndrome/acute myeloid leukemia (43). IL-8 could also cause the pathology of platelets, leading to platelet hyper-activation and spreading (44).

Another important proinflammatory cytokine, IL-1β, was also shown to be increased in pSS-associated thrombocytopenia compared to general pSS in our study. IL-1β can be induced by activation of the TLR7 signaling pathway (45). At the peripheral blood level, patients with pSS showed higher expressions of IL-1β than healthy controls, and the IL-1β level was related to disease damage (46). As for thrombocytopenia, studies revealed that IL-1β together with IL-6 and IL-8, could increase hypercoagulability of whole blood and induce platelet hyper-activation and spreading (44). Meanwhile, the complement-IL-1β loop could cause impairment of mesenchymal stem cells, leading to immune thrombocytopenia (47).

In patients with pSS-associated thrombocytopenia, no previous studies have reported the role of IL-1β or IL-8. According to our findings, platelets showed a negative correlation with serum levels of IL-1β and IL-8. Thus, IL-8 may be a promising biomarker to identify pSS-associated thrombocytopenia in general patients with pSS. It whould be helpful for monitoring disease progression and defining severity of this disease in the clinic. However, both IL-1β and IL-8 may also reveal disease activity of pSS-associated thrombocytopenia, but this still requires further study.

These findings have practical implications. Based on our findings, intervention in the TLR signaling pathway may help identify new treatment methods for pSS-associated thrombocytopenia. In our study, the TLR signaling pathway was the most significant pathway for pSS-associated thrombocytopenia, with the highest number of DEGs in the KEGG enrichment analysis. Hydroxychloroquine (HCQ) is widely used for autoimmune diseases via suppressing TLR signaling pathway. Torigoe et al. (48) found HCQ could markedly suppress the TLR9-mediated human B cell functions during inflammatory processes, including B cell subset differentiation, IgG production, and cytokines secretion. However, HCQ is a weak drug as a monotherapy in pSS associated thrombocytopenia treatment in our experience. We suspect that the immune-modulatory effect of HCQ on TLR signaling pathway may not be strong enough in this condition. Recently, new studies have focus on the intervention of several proteins in the TLR signaling pathways (e.g., IKK-2 and MyD88), which have been identified as potential therapeutic targets for the treatment of certain diseases [e.g., systemic lupus erythematosus (SLE), rheumatoid arthritis (RA), etc.] (49). Recently, new studies have focused on the involvement of several proteins in the TLR7 signaling pathways (e.g., TLR7 and MyD88), which have been identified as potential therapeutic targets for the treatment of autoimmune diseases (49). Inhibition of TLR7 represents a potential therapeutic strategy to reduce anti-RNA autoantibody production and attenuate glomerulonephritis in lupus mice (50). Inhibition of the TLR7 pathway led to platelet count growth in the pSS mouse model in our study. Therefore, strategies inhibiting the TLR7 pathway may benefit patients with pSS-associated thrombocytopenia, which should be a major focus for further investigation in the clinic.

There are some limitations to our study. First, the limited sample number may have affected the power of this study to detect differences between divergent groups. Second, there is currently no standard animal model for pSS-associated thrombocytopenia. Meanwhile, as a more rigorous experimental design, B cell specific TLR7 knockout mice model should be used. These complex mechanisms need to be intensively investigated in the future.

## Conclusion

In conclusion, the present study showed that pSS-associated thrombocytopenia might be a subset characterized by a systemic inflammatory state. The TLR7 signaling pathway was upregulated in patients with thrombocytopenia compared with that in patients without pSS. IL-1β and IL-8 might be promising biomarkers for this complication. TLR7 pathway regulation might be a potential new treatment option for patients with pSS-associated thrombocytopenia.

## Data Availability Statement

The datasets presented in this study can be found in online repositories. The names of the repository and accession number can be found below: http://www.ncbi.nlm.nih.gov/bioproject/703047.

## Ethics Statement

The studies involving human participants were reviewed and approved by Ethics Committee of PUMCH. The patients/participants provided their written informed consent to participate in this study. The animal study was reviewed and approved by Ethics Committee of PUMCH.

## Author Contributions

SZ, JQ, LW, and ML designed the study. SZ, LW, ML, DX, YZ, FZ, and XZ recruited patients. SZ and JQ performed the experiments, analyzed the data, and wrote the manuscript. All authors contributed to the article and approved the submitted version.

## Conflict of Interest

The authors declare that the research was conducted in the absence of any commercial or financial relationships that could be construed as a potential conflict of interest.

## Supplementary Material

Supplementary Figure 1 DEGs in pSS associated thrombocytopenia. **(A)** The hierarchical clustering heat maps of DEGs in group 1. **(B)** The hierarchical clustering heat maps of DEGs in group 2. **(C)** Volcano plot of DEGs in group 1. **(D)** Volcano plot of DEGs in group 2. **(E)** Venn diagram showing the number of DEGs in group 1 and group 2, with an overlap of 19 DEGs. DEGs, differentially expressed genes. * group 1: pSS associated thrombocytopenia patients and healthy controls. group 2: pSS associated thrombocytopenia patients and pSS patients.

Supplementary Figure 2 Pathway analysis of 19 differentially expressed genes (DEGs) in pSS associated thrombocytopenia. **(A)** Bar-plot of relevant and significantly enriched gene sets from KEGG data bases: each bar of KEGG terms represent DEGs counts enriched. **(B)** KEGG pathway network analysis: The large nodes in the network represent the KEGG terms, and the small nodes are DEGs. **(C)** KEGG mapper of toll-like receptor singaling pathway: red colors represent DEGs. DEGs, differentially expressed genes; KEGG, Kyoto Encyclopedia of Genes and Genomes; GO, gene ontology.

Supplementary Figure 3 Visualization of PPI network and hub genes using cytoscape. **(A)** PPI network was identified for 13/19 DEGs: The color represents the degree of the nodes, and the darker the edge displayed, the higher the degree of interaction is. **(B)** Model PPI network originated from a with most significant interactions (MCODE score > 6). **(C)** Interaction network of hub genes. **(D)** The co-citation network of hub genes, the number on the line represents the number of studies co-cited. DEGs, differentially expressed genes; PPI, protein-protein interaction.

Supplementary Figure 4 Toll-like receptor (TLR) pathway intervention-induced expression of the TLR7 pathway and changes in the related serum markers in NOD mice. Peripheral blood or serum was obtained from NOD mice at the age of 13 weeks, 5 weeks after intervention of CA-4948/Resiquimod/saline. **(A)** mRNA relative expression levels TLR7, MyD88, IRAK4, TRAF6, and NF-κB. **(B)** mRNA relative expression levels IL-1β, IL-8, thrombopoietin (TPO), and Megakaryocyte Colony Stimulating Factor (MK-CSF) in peripheral blood. **(C)** Serum levels of IL-1β, IL-8, TPO, and ML-CSF. Compared with the control group, treatment of NOD mice with CA-4948 led to a significant decrease in serum and peripheral blood mRNA expression of the TLR7 pathway molecules, IL-1β, IL-8, and MK-CSF levels and increase in serum TPO levels (^*^*p* < 0.05, ^**^*p* < 0.01, ^***^*p* < 0.001; ns, not significant).

Supplementary Table 1 Real-time polymerase chain reaction primer sequences.

Supplementary Table 2 Expression (log FC) of DEGs in pSS-associated thrombocytopenia.
