# Supplementary figures and images for: Activation of Toll-Like Receptor 7 Signaling Pathway in Primary Sjögren's Syndrome-Associated Thrombocytopenia
Source: Front Immunol. 2021 Mar 9;12:637659. doi: 10.3389/fimmu.2021.637659 (PMC7986855; doi:10.3389/fimmu.2021.637659)

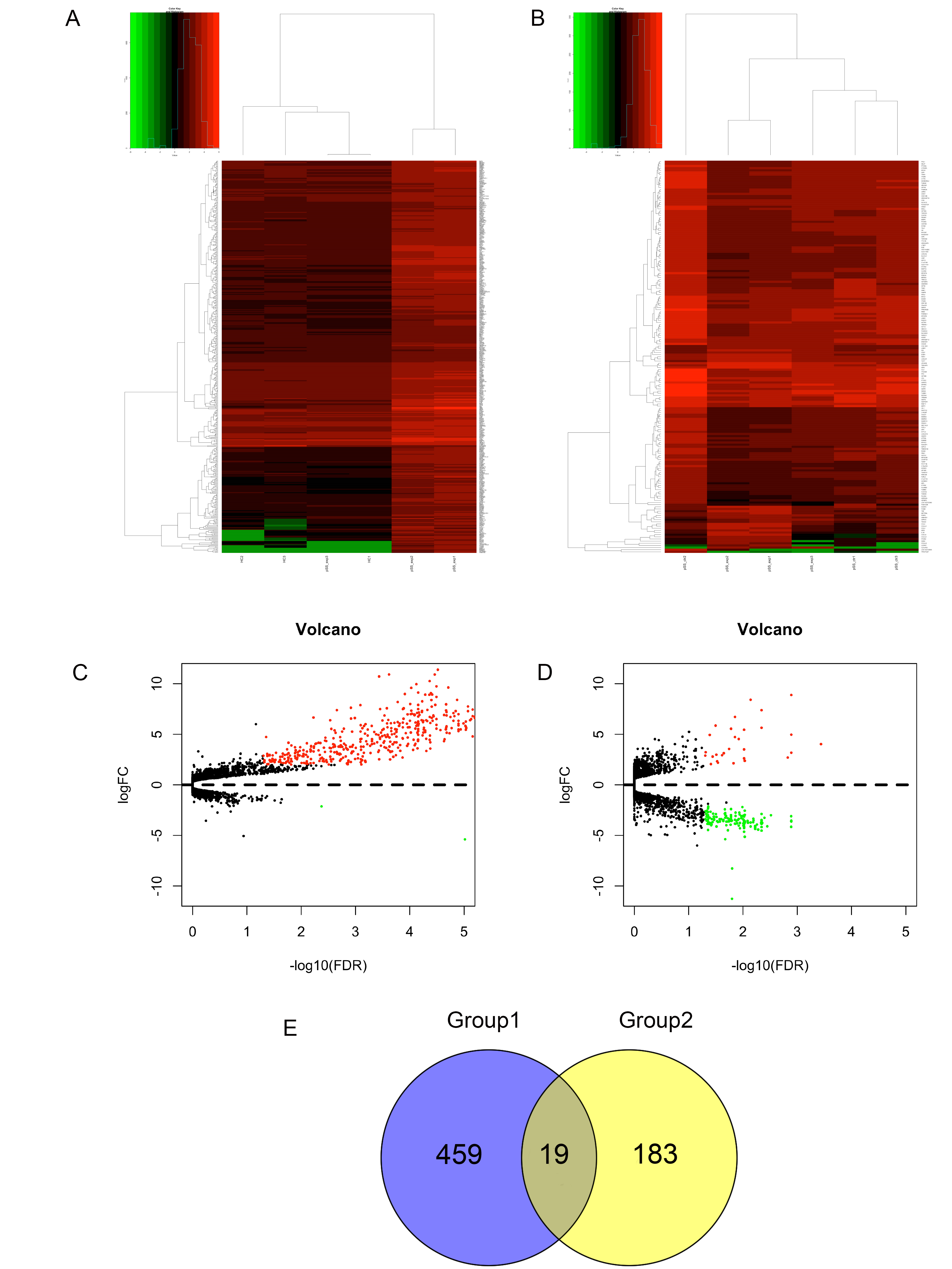

Supplement: Supplementary Figure 1 — DEGs in pSS associated thrombocytopenia. (A) The hierarchical clustering heat maps of DEGs in group 1. (B) The hierarchical clustering heat maps of DEGs in group 2. (C) Volcano plot of DEGs in group 1. (D) Volcano plot of DEGs in group 2. (E) Venn diagram showing the number of DEGs in group 1 and group 2, with an overlap of 19 DEGs. DEGs, differentially expressed genes. * group 1: pSS associated thrombocytopenia patients and healthy controls. group 2: pSS associated thrombocytopenia patients and pSS patients. [file Image_1.JPEG]

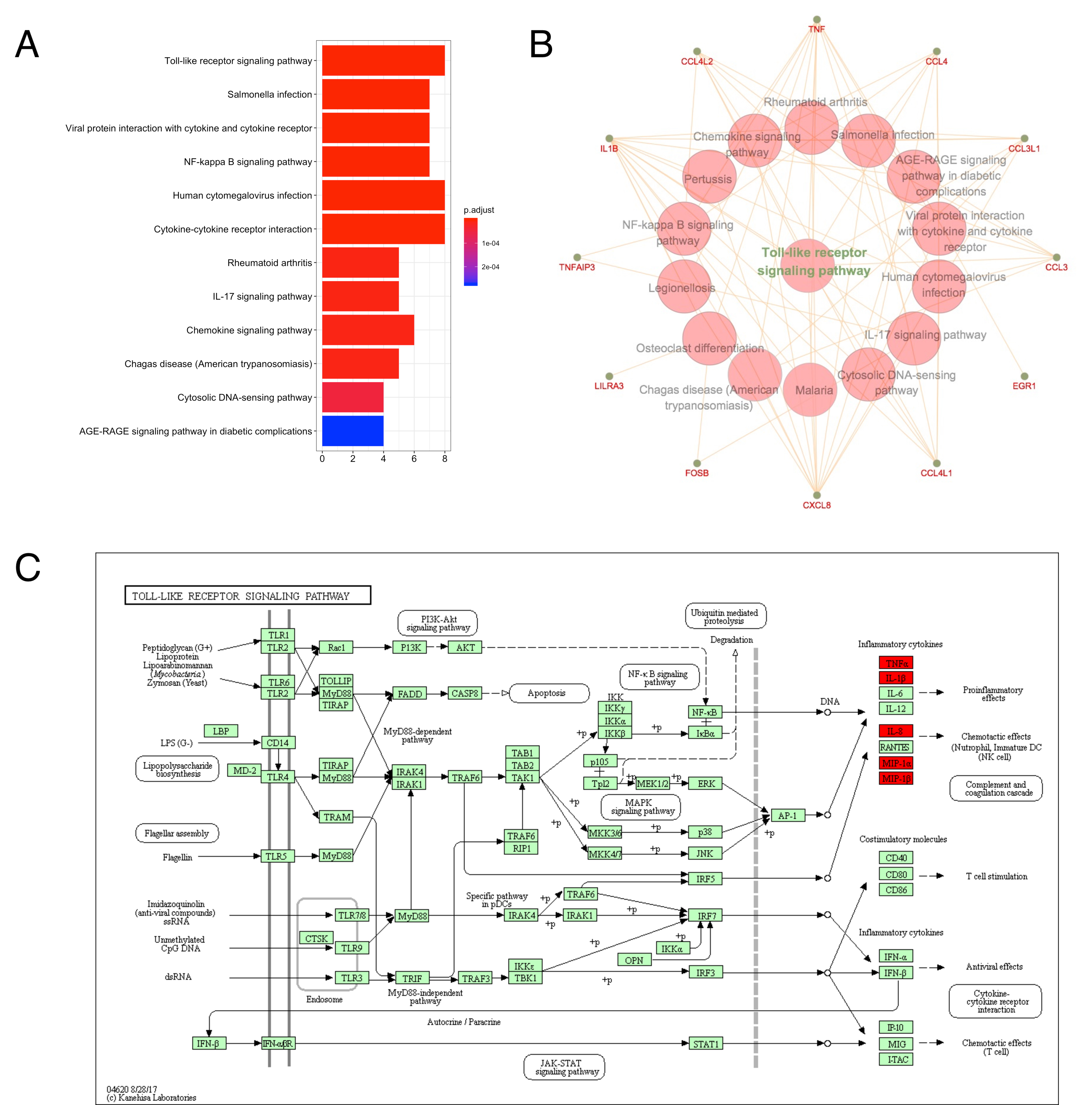

Supplement: Supplementary Figure 2 — Pathway analysis of 19 differentially expressed genes (DEGs) in pSS associated thrombocytopenia. (A) Bar-plot of relevant and significantly enriched gene sets from KEGG data bases: each bar of KEGG terms represent DEGs counts enriched. (B) KEGG pathway network analysis: The large nodes in the network represent the KEGG terms, and the small nodes are DEGs. (C) KEGG mapper of toll-like receptor singaling pathway: red colors represent DEGs. DEGs, differentially expressed genes; KEGG, Kyoto Encyclopedia of Genes and Genomes; GO, gene ontology. [file Image_2.JPEG]

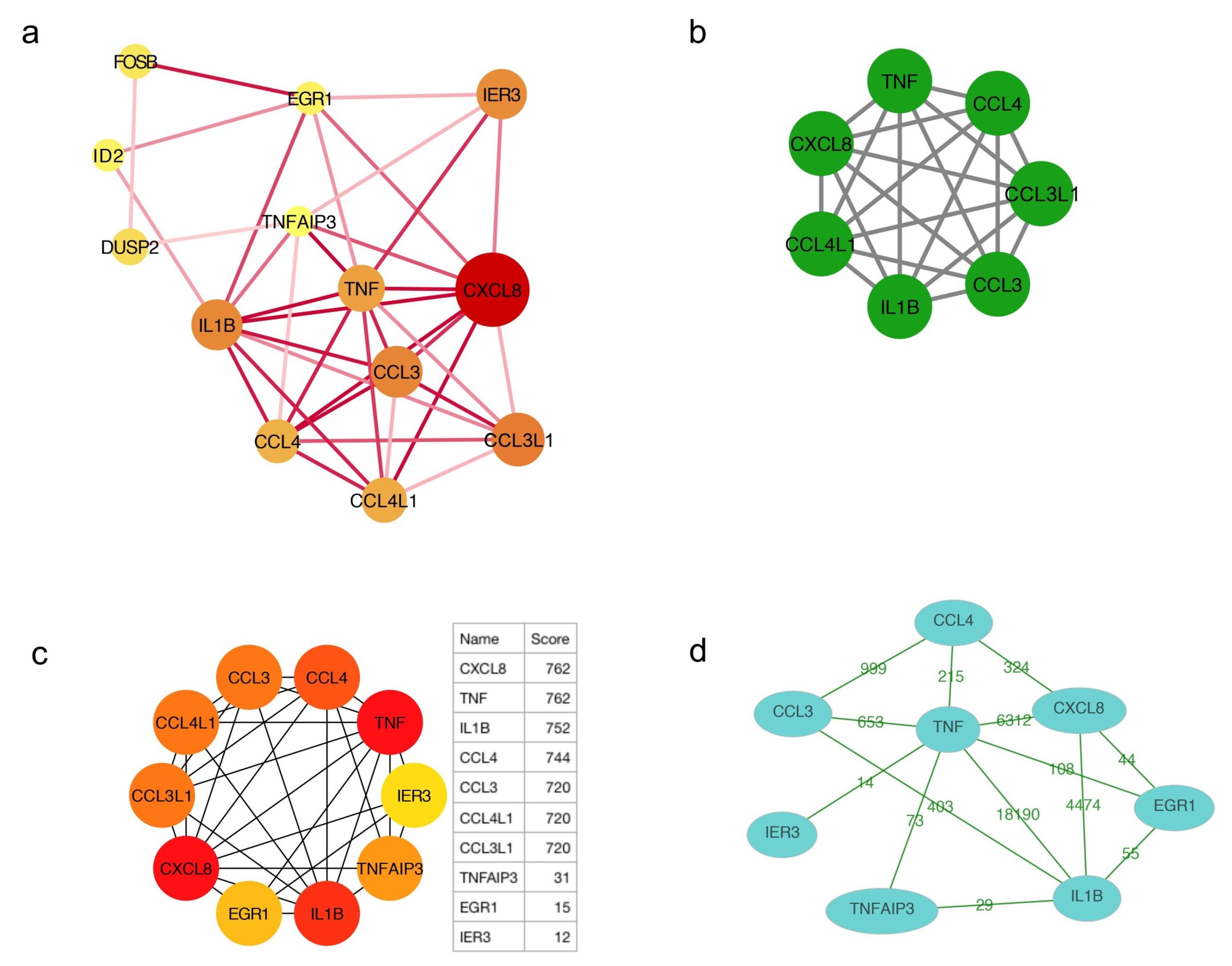

Supplement: Supplementary Figure 3 — Visualization of PPI network and hub genes using cytoscape. (A) PPI network was identified for 13/19 DEGs: The color represents the degree of the nodes, and the darker the edge displayed, the higher the degree of interaction is. (B) Model PPI network originated from a with most significant interactions (MCODE score > 6). (C) Interaction network of hub genes. (D) The co-citation network of hub genes, the number on the line represents the number of studies co-cited. DEGs, differentially expressed genes; PPI, protein-protein interaction. [file Image_3.JPEG]

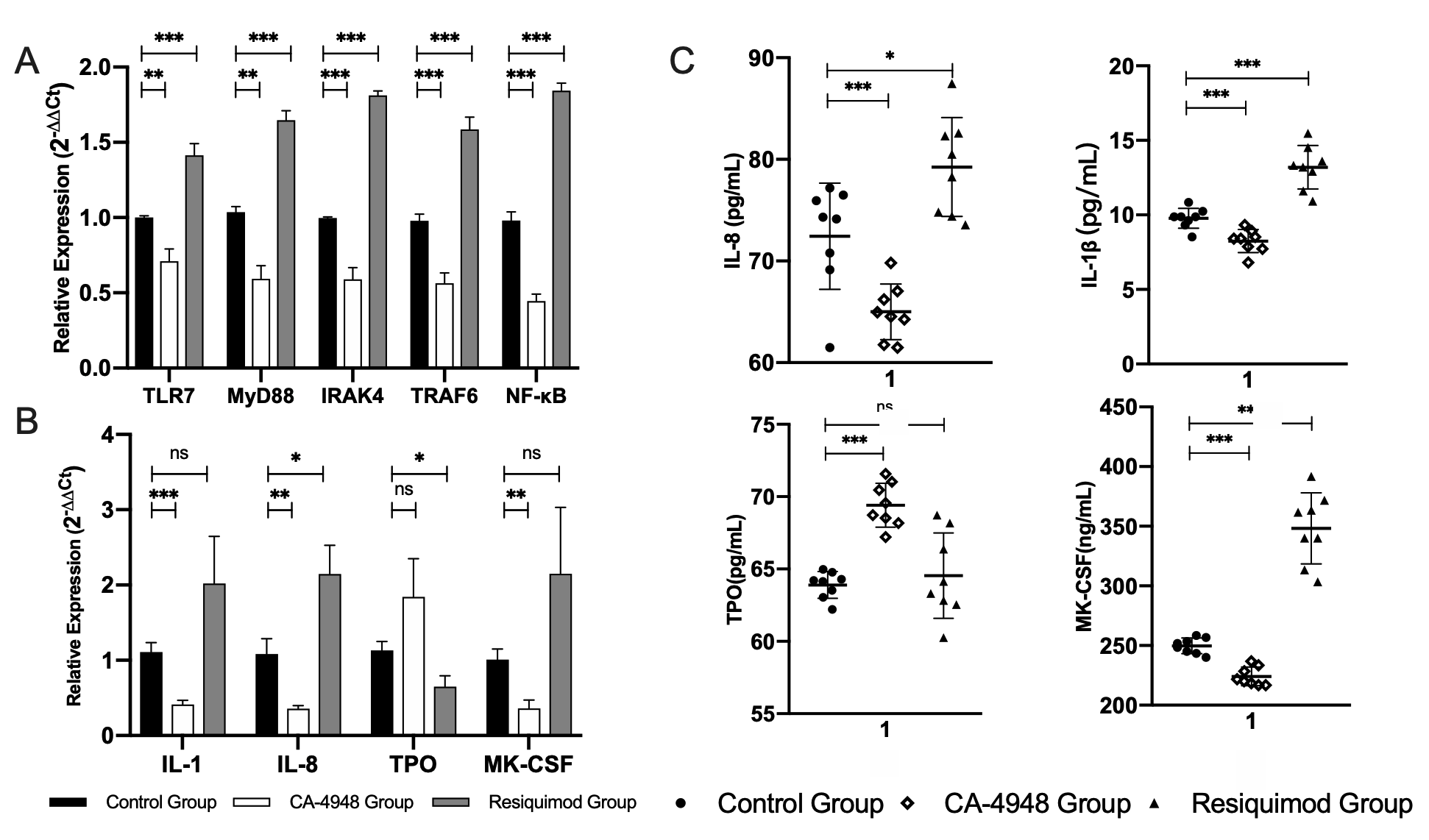

Supplement: Supplementary Figure 4 — Toll-like receptor (TLR) pathway intervention-induced expression of the TLR7 pathway and changes in the related serum markers in NOD mice. Peripheral blood or serum was obtained from NOD mice at the age of 13 weeks, 5 weeks after intervention of CA-4948/Resiquimod/saline. (A) mRNA relative expression levels TLR7, MyD88, IRAK4, TRAF6, and NF-κB. (B) mRNA relative expression levels IL-1β, IL-8, thrombopoietin (TPO), and Megakaryocyte Colony Stimulating Factor (MK-CSF) in peripheral blood. (C) Serum levels of IL-1β, IL-8, TPO, and ML-CSF. Compared with the control group, treatment of NOD mice with CA-4948 led to a significant decrease in serum and peripheral blood mRNA expression of the TLR7 pathway molecules, IL-1β, IL-8, and MK-CSF levels and increase in serum TPO levels (*p < 0.05, **p < 0.01, ***p < 0.001; ns, not significant). [file Image_4.TIFF]
