# Supplementary material for: Activation of Toll-Like Receptor 7 Signaling Pathway in Primary Sjögren's Syndrome-Associated Thrombocytopenia
Source: Front Immunol. 2021 Mar 9;12:637659. doi: 10.3389/fimmu.2021.637659 (PMC7986855; doi:10.3389/fimmu.2021.637659)
Supplement: Supplementary Table 1 — Real-time polymerase chain reaction primer sequences. [file Table_1.DOCX]

**sTable 1 Real-Time PCR primer sequences**

| **Species** | **Target Name** | **Primer** | |
| --- | --- | --- | --- |
| Human | Actin | Actin-F | GACAGGATGCAGAAGGAGATTACT |
|  |  | Actin-R | TGATCCACATCTGCTGGAAGGT |
|  | IL-1 | IL-1-F | TTTGAGTCTGCCCAGTTCCC |
|  |  | IL-1-R | GTTATATCCTGGCCGCCTT |
|  | IL-8 | IL-8-F | CAGTTTTGCCAAGGAGTGC |
|  |  | IL-8-R | TAATTTCTGTGTTGGCGCAGT |
|  | TPO | TPO-F | ACACGCACTGGCACTAAATCCAC |
|  |  | TPO-R | CACTCTCCTGCTCCGAGTCCT |
|  | MK-CSF | MK-CSF-F | GCCTGCTGTGGACTTTAGCTT |
|  |  | MK-CSF-R | CCTCCAGCAGAAGGGTCACT |
|  | TLR7 | TLR7-F | CCTAAGTGGAAATTGCCCTC |
|  |  | TLR7-R | CTTTTAATTCTGTCAGCGCAT |
|  | TRAF6 | TRAF6-F | ATGCGGCCATAGGTTCTGC |
|  |  | TRAF6-R | TCCTCAAGATGTCTCAGTTCCAT |
|  | MyD88 | MyD88-F | GCGCCGCCTGTCTCTGTTCTTG |
|  |  | MyD88-R | CAAAGTCCATCTCCTCCGCCAGC |
|  | IRAK4 | IRAK-F | ATAACTTTGATGAACGACCC |
|  |  | IRAK-R | GTTGTGTTATTTACGTAGCCT |
|  | NF-κB p65 | NF-κB p65-F | AGTCAGCGCATCCAGACCAAC |
|  |  | NF-κB p65-R | GGTCCCGCACTGTCACCT |
| Mice | Actin | Actin-F | CCAGCCTTCCTTCTTGGGTA |
|  |  | Actin-R | CAATGCCTGGGTACATGGTG |
|  | IL-1 | IL-1-F | TTGAAGTTGACGGACCCCAA |
|  |  | IL-1-R | CCACAGCCACAATGAGTGA |
|  | IL-8 | IL-8-F | GGCCCAATTACTAACAGGT |
|  |  | IL-8-R | ATAGAGGCTTTTCATGCTCA |
|  | TPO | TPO-F | TCCTTGCTTCCCCATACAGCTTC |
|  |  | TPO-R | GGTTGCCAAAGAGAGCACCT |
|  | MK-CSF | MK-CSF-F | CTCTAGCCGAGGCCATGTGGA |
|  |  | MK-CSF-R | CTCTAGCCGAGGCCATGTGGA |
|  | TLR7 | TLR7-F | TCTCTCCAGATTCCTTCCGTA |
|  |  | TLR7-R | AATCTGCAGCCTCTTGGTA |
|  | TRAF6 | TRAF6-F | CAAACAGTTGGAGAGTCGCCTA |
|  |  | TRAF6-R | CGTTTGAGCTCGCCCACGTA |
|  | MyD88 | MyD88-F | GTCCATTGCCAGCGAGCTA |
|  |  | MyD88-R | AAAGTCCTTCTTCATCGCCTT |
|  | IRAK4 | IRAK4-F | CGGGGAGAAATAACACCCAA |
|  |  | IRAK-R | TAATATCCAGCAGTAGTTGAGGT |
|  | NF-κB p65 | NF-κB p65-F | CCCTGTCCTCTCACATCCGA |
|  |  | NF-κB p65-R | CACAGCAAGAAGATCTCATCCCC |
