# Supplementary material for: Activation of Toll-Like Receptor 7 Signaling Pathway in Primary Sjögren's Syndrome-Associated Thrombocytopenia
Source: Front Immunol. 2021 Mar 9;12:637659. doi: 10.3389/fimmu.2021.637659 (PMC7986855; doi:10.3389/fimmu.2021.637659)
Supplement: Supplementary Table 2 — Expression (log FC) of DEGs in pSS-associated thrombocytopenia. [file Table_2.DOCX]

**sTable.2 Expression (log FC) of DEGs in pSS associated thrombocytopenia**

| Gene | LogFC  in group2 | FDR  in group 2 | LogFC  in group1 | FDR  in group 1 |
| --- | --- | --- | --- | --- |
| TNF | 4.96 | 1.29E-03 | 4.55 | 4.98E-05 |
| CXCL8 | 8.88 | 1.29E-03 | 9.74 | 3.23E-05 |
| CCL3 | 5.65 | 4.54E-03 | 5.61 | 1.70E-05 |
| G0S2 | 7.38 | 4.54E-03 | 12.33 | 1.09E-05 |
| LILRA3 | 8.42 | 7.23E-03 | 10.26 | 4.31E-05 |
| IER3 | 5.44 | 9.53E-03 | 7.71 | 2.98E-06 |
| DUSP2 | 3.50 | 9.53E-03 | 3.91 | 8.12E-05 |
| TNFAIP3 | 2.63 | 9.53E-03 | 2.24 | 1.36E-03 |
| CCL4 | 4.53 | 1.19E-02 | 5.42 | 3.35E-06 |
| CCL4L2 | 6.72 | 1.40E-02 | 8.92 | 5.19E-05 |
| CCL4L1 | 4.72 | 1.40E-02 | 5.94 | 3.94E-06 |
| IL1B | 5.54 | 1.66E-02 | 10.23 | 3.27E-06 |
| METRNL | 3.55 | 1.80E-02 | 4.02 | 2.08E-04 |
| ID2 | 2.93 | 2.43E-02 | 3.78 | 6.57E-03 |
| PER1 | 2.33 | 2.99E-02 | 2.42 | 7.68E-04 |
| EGR1 | 2.98 | 3.09E-02 | 2.93 | 1.80E-04 |
| CCL3L1 | 5.86 | 3.20E-02 | 6.66 | 5.94E-03 |
| FFAR2 | 4.94 | 4.09E-02 | 8.40 | 1.34E-05 |
| FOSB | 3.23 | 4.86E-02 | 3.49 | 1.39E-03 |
